# Designing and Developing a Mobile Smartphone Application for Women with Gestational Diabetes Mellitus Followed-Up at Diabetes Outpatient Clinics in Norway

**DOI:** 10.3390/healthcare3020310

**Published:** 2015-05-21

**Authors:** Lisa Maria Garnweidner-Holme, Iren Borgen, Iñaki Garitano, Josef Noll, Mirjam Lukasse

**Affiliations:** 1 Faculty of Health Sciences, Oslo and Akershus University College of Applied Sciences, PB 4 St. Olavs plass, Oslo N-0130, Norway; E-Mails: iren.borgen@hioa.no (I.B.); mirjam.lukasse@hioa.no (M.L.); 2 University Graduate Centre, P.O. Box 70, Kjeller N-2027, Norway; E-Mails: igaritano@garitano.org (I.G.); josef@unik.no (J.N.)

**Keywords:** gestational diabetes mellitus, mobile health care, healthy eating, physical activity, user-centered design

## Abstract

The prevalence of Gestational Diabetes Mellitus (GDM) is increasing worldwide. Controlling blood sugar levels is fundamental to the management of GDM. Current practice in Norway includes patients registering blood sugar levels in a booklet and receiving verbal and/or written health information. A smartphone application may provide patients individually targeted and easily available advice to control blood sugar levels. The aim of this paper is to document the process of designing and developing a smartphone application (the Pregnant+ app) that automatically transfers blood sugar levels from the glucometer and has information about healthy eating and physical activity. This formative research included expert-group discussions among health professionals, researchers and experts in data privacy and security. User-involvement studies were conducted to discuss prototypes of the app. Results indicated that the content of the application should be easy to understand given the varying degree of patients’ literacy and in line with the information they receive at clinics. The final version of the app incorporated behavior change techniques such as self-monitoring and cues to action. Results from the first round of interactions show the importance of involving expert groups and patients when developing a mobile health-care device.

## 1. Introduction

The prevalence of Gestational Diabetes Mellitus (GDM), defined as glucose intolerance with onset or first recognition during pregnancy [1], is increasing worldwide. Prevalence rates of GDM in population-based studies vary from 1%–22% [2]. This great range might be due to variability in screening diagnostics and the heterogeneity of study populations [3]. Maternal obesity, advanced maternal age, a family history of diabetes, a previous history of GDM and belonging to certain ethnic groups increase the risk of developing GDM [4]. Women of Asian origin, and to a lesser degree those of African origin, tend to develop GDM at a lower body mass index and younger age compared to other ethnic groups [5]. The development of GDM may lead to long-term consequences for maternal and offspring health [6].

Controlling blood sugar levels is fundamental to the management of GDM [6]. Healthy eating, physical activity and weight management help patients to have stable blood sugar levels without medical treatment [7]. In Norway, national guidelines for diagnosis and treatment of diabetes outline screening criteria for GDM [8]. Women with a 75 g oral glucose tolerance test (OGTT) ≥9.0 mmol/L (2-h plasma glucose) receive specialized care at diabetes outpatient clinics. At most of these clinics, women are encouraged to register their blood sugar levels four times a day (once fasting and three times 1.5 h post prandial). Levels are registered in a booklet and discussed with the health professionals approximately every two weeks. Women receive further verbal health information accompanied by leaflets

Pregnant women, especially those with GDM, may be easy to reach with health-related information due to regular contact with health professionals and motivated for behavior change [9,10]. However, previous research indicates that pregnant women can have problems processing the health information they receive in antenatal care [11]. In addition, researchers found poor knowledge about GDM and lack of awareness that GDM increases the risk of disease long term [12,13,14]. Health professionals, on the other hand, may find it challenging to communicate about diet and physical activity, especially when faced with a multicultural and socially diverse population [15,16].

A smartphone application (app) may provide a novel way to provide health information and may assist in blood sugar control for women with GDM. In Norway, approximately 81% of the adult population has a smartphone [17]. Smartphone apps as medical devices are new tools in the management of type 1 and 2 diabetes [18]. Women with GDM may have other informational needs than patients with type 1 or 2 diabetes. Even though mobile technology can have many advantages for providing health information, its limitations must be considered in order to meet the needs of target groups [19].

Given the increase in available smartphone apps, it is important to document the process designing and developing an app in order to enhance trustworthiness of the mobile health care device. We found several smartphone apps specifically for women with GDM [20]. However, none of the apps supported self-monitoring of blood sugar levels available for both Android and iOS and strict privacy requirements.

## 2. Experimental Section

**Figure 1:**
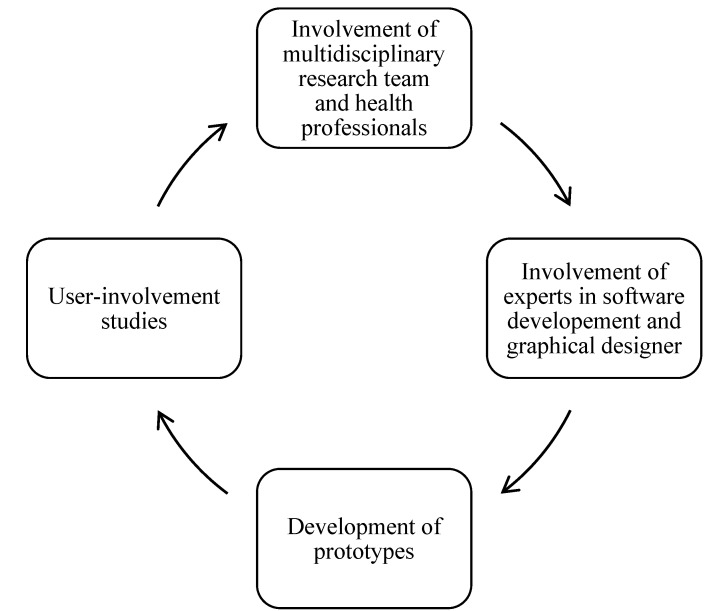
Iterative process of the designing and developing the Pregnant+ app.

Important criteria for successful medical smartphone apps are content quality, usability, the need to match apps to consumers’ literacy levels, app security and user privacy [19]. To meet these criteria, design and development of our smartphone app involved a multidisciplinary research team, experts in software development, data privacy and security, a graphical user interface designer and language editor, and health professionals and patients at five diabetes outpatient clinics in the area of Oslo, Norway. Figure 1 illustrates the iterative process of the design and development of the Pregnant+ app (Figure 1).

### 2.1. Involvement of Researchers and Health Professionals

Initial brainstorming with researchers and health professionals was conducted in April 2014, followed by discussions in specific groups. The multidisciplinary research team included researchers with experience in software development, data privacy and security, obstetrics and midwifery and nutrition and physical activity. The first step in designing the app was to discuss main content themes and technical functions. Thereafter, discussions continued in specified research groups focusing on software development, health communication strategies and nutrition and physical activity related to GDM. Content quality is one of the most important aspects for reliable health apps [19]. We discussed the content of the app in the expert groups and reviewed the literature to assure the quality of the health information provided in the app. Information about GDM, healthy eating and physical activity was derived from national and international guidelines [21,22,23,24]. The main messages for healthy eating were to eat regularly, limit the intake of sugar and increase the intake of vegetables and whole grain. In addition, we reviewed the literature to identify relevant health communication theories on interventions by smartphones [25].

Lack of medical professional involvement in the design of health apps may undermine the content quality of those apps [19]. The review by Arnold *et al.* found that most of the mobile apps for diabetics lacked involvement of health professionals [26]. Health professionals were regularly involved in development of our app, had very positive attitudes toward it and contributed their knowledge.

### 2.2. Application Development

In order to guarantee a user-friendly interface and a highly automated smartphone app, the Pregnant+ app graphical user interface and features were designed by a professional designer and experts in software development, respectively.

The app has three main innovative features: (I) privacy, no medical data leaves the device; (II) application self-update, the ability to update app content without having to update the entire app and (III) simple, automated transfer of glucometer readings.

To address the needs of the Norwegian market the Pregnant+ app was developed for smartphones running two of the most popular Operating Systems (OSs), Android and iOS [27]. In addition, the most popular versions of both OSs were considered. Thus, in the case of Android, the Pregnant+ app is compatible with versions 4.1× and higher [28]. For iOS, the app supports smartphones running version 8 [29].

#### 2.2.1. Software Development Challenges

One of our main challenges was to develop an interface between the smartphone app and a glucometer to allow users to automatically transfer blood sugar measurements. Only a few smartphone app offer direct data transfer from glucometers [18].

Considering the increasing number of Bluetooth-enabled glucometers, and focusing on a development of a wireless, automated data-transfer process, the Bluetooth protocol was selected. Women participating in the randomized controlled trial, that will be conducted to test the app, will receive a blood glucose meter with incorporated Bluetooth function (Diamond Mini, Fora Care Inc., Moorpark, CA, USA). The Bluetooth protocol enables portable electronic devices to connect and communicate wirelessly [28].

Interviews revealed that some users were concerned about privacy of their personal health information registered in the app. In a society where personal data can be analyzed by Big Data techniques, there is increasing interest by companies that see personal data as a valuable asset [29]. A majority of companies provide free cloud services and force their clients to use them with the proviso that clients must allow the company to analyze personal data, feathering their nests with the results. Such cloud storage of personal medical data was not favored by our national data privacy advisors, and therefore it was not considered. Our approach of privacy-preserving glucose measurements results from intensive discussions with the Norwegian Data Protection Authority and the Privacy Responsible for the Public Health. The main focus was encrypted storage, to provide the user with the authority to deal with the data, and avoid glucose level data leaving the phone. The glucose measurement data are encrypted and stored in the mobile phone itself. We considered loss of the smartphone, and evaluated extra security against usability. Given the advice from the health personnel, we avoided an extra authentication of the app, and focused on device authentication. In case a smartphone is lost, the user will ask for access to the app on a new device. As only one device per user is white listed, the new device will disable the old device, reducing the app to a generic information page. In addition, some participants perceived the storage of their glucose levels on the smartphone as more secure than their current registration in a booklet.

Another challenge was the lack of Application Programming Interface (API) to directly communicate with blood-sugar measuring devices, which forced us to develop our own APIs. Glucometer vendors provide their devices with an app for iOS and Android phones. However, these apps involve the usage of a cloud service to store and analyze data. Even the API provided by vendors, that allows third parties to develop their own apps, make usage of cloud services. Thus, the development of the Pregnant+ app involved the development of a glucometer communication protocol, allowing that the app automatically detects and retrieves measurements from a Bluetooth-based glucose-monitoring system without an external cloud system.

To protect user privacy, the Pregnant+ app was designed to store data locally and prevent it from being shared with any other smartphone app. In addition, the app has the option to delete glucose values older than a selected time, e.g., older than 6 weeks, 2 months, or 3 months. Background for this decision is the privacy-awareness discussed earlier, and the time-interval at which patients visit their doctor. We added two weeks of uncertainty to ensure that at least 6 weeks of data are available to be discussed with the doctor. Furthermore, the app contains an information page that fully informs users what information has been gathered and gives the purpose of the study.

One of the main challenges of getting smartphone apps into app stores quickly is the time delay that app stores, such as Apple and Google, impose due to pre-release testing of apps. Furthermore, each time the app is updated, it must be tested again, again delaying its availability. In addition, the app update process requires user interaction and could cause loss of some glucose measurements. In order to avoid such problems, the Pregnant+ app caches online content in the device. When a user accesses an information page in the app, the information is checked against an online repository and updated if out of date. This process obviates the need to update the entire app every time updates are available.

Once an app is published in the corresponding app marketplace, it is available for everyone who wants to download and install it. However, our app will first be tested in a randomized controlled trial. Thus, the app’s full content will be visible only to participants of the intervention group. Participants of the control group will be able to download the app but will have access to restricted content only. Consequently, an access control was implemented. During the first run, this access control asks for and checks each participant phone number against a remote database. The phone number was first requested when the participant entered the research study, and it is used only to limit app content and send a Short Message Service (SMS) after the study is finished. All participants are informed about the use of their phone number during presentation of the Pregnant+ application.

Rather than allowing electronic exchange of sensitive health data, we implemented a print function that allows patients to share their glucose measurements with health professionals. The Pregnant+ application allows users to print glucose measurements in a format known by their health professionals.

### 2.3. User-Involvement

A review of mobile apps for diabetics concluded that several apps lacked suitability and usability for their main target group [26]. User-involvement may be described as an iterative development process that engages users in conceptualization, design and final production of a smart phone mobile app [30,31]. Involvement of users from the beginning of the development process has been shown to increase appeal and user-friendliness of mobile app [30]. Possible methods to incorporate user-involvement in design are interviews, surveys or observations [31].

During design and development of our app, patients were involved in two stages. Participants were purposively recruited at three outpatient clinics in the area of Oslo, Norway. The interviews were carried out at the outpatient clinics by the first author, a public health nutritionist, and occasionally accompanied by other project members. Inclusion criteria for participating in the study were a diagnosis of GDM, no need for medicamental treatment of diabetes, and experience in registering blood sugar levels. Almost all of the approached women (*n* = 22) participated in one of the user-involvement studies. The first stage of the user-involvement study (*n* = 10) was performed between May and July 2014. Interviews involved initial questions about the use of smartphone app and presentation of the first Pregnant+ prototype on a tablet. The second stage was conducted between December 2014 and January 2015. In the second stage, users were asked to speak aloud while performing given tasks. This is a well-known method of user-involvement [31]. All interviews were audio-taped and transcribed *verbatim*. A quotation count report (Atlas.ti version 6.2.15, ATLAS.ti Scientific Software Development GmbH, Berlin, Germany, 2011) was performed to analyze the interviews. The Norwegian Social Science Data Service approved the study, and participants gave informed consent.

## 3. Results and Discussion

### 3.1. Group Discussions among Researchers and Health Professionals

Initial discussions in the experts’ group outlined the importance of individualization and visualization in the app. Individualization was considered important given the heterogeneous group of patients. This led to the introduction of the My Profile feature, which will be described in a following section. Efforts were made to visualize healthy eating and physical activity in order to overcome language barriers. The overall strategy was to visualize cues to actions related to healthy eating and physical activity in order to illustrate how patients can change behaviors.

Health professionals stated that the content of the app needed to be more specific to GDM and its consequences. They also stressed that optimal blood sugar levels can be highly individual. A primary challenge for the five participating clinics was to agree upon optimal blood glucose levels during pregnancy given the current lack of national guidelines.

### 3.2. Interviews and Task Performance among Patients

In total, 21 pregnant women were involved in development of the app between June 2014 and January 2015. Participants varied in terms of ethnicity, literacy level and knowledge about GDM. Table 1 shows characteristics of study participants.

Even though only 9 participants considered themselves frequent users of apps, all (*n* = 21) stated that they would use the Pregnant+ app regularly. As compared to their current routines, they made such statements as:

*“I think it is very good. You get inspired to do things right. It’s pretty easy, yes, because you have your phone with you all the time. I always forget about the registration booklet and have to find a pen.”* 

**Table 1:** Ethnicity and previous diagnosis of Gestational Diabetes Mellitus (GDM) of participants (*n* = 21).

Characteristics		Number of Participants	Number of Participants
		User-Involvement Study 1	User-Involvement Study 2
Region of origin	Norway	4	6
	Africa	0	2
	Asia	4	1
	Eastern-Europe	1	1
	South-America	1	1
Previous diagnosis of GDM	Never	6	10
	Once	3	0
	Twice	1	1

Patients perceived that the app would make it easier to register and control their blood sugar levels. We found that many participants did not know their optimal blood sugar levels. They valued that the app provides real-time feedback on their blood sugar levels, demonstrated by a graph that will appear on the smartphone after the automatic transfer or manual registration of the levels. Several participants appreciated that the app combines both registration of blood sugar levels and information about nutrition, physical activity and GDM, thus providing a novel tool compared to their current registration in booklets.

#### 3.2.1. Initial Interviews

Table 2 summarizes feedback from users and resulting adjustments to the app.

**Table 2:** User feedback on the initial Pregnant+ prototype.

Theme of Feedback	Quote	Adjustments to the App
Use of language	*“What is whole grain”*	Use easier language; avoid jargon; develop a diabetes lexicon in the application.
	*“What is tests”*	
Conflicting information compared to advice from health professionals	*“But, here at the clinic, they told me that I should not eat too much rice”*	Standardize app content with content of leaflets from diabetes outpatient clinics.
Information about gestational diabetes	*“Women need more risk awareness. They think that GDM disappears after delivery”.*	Add more information about the risks of having GDM and future health consequences for mother and child.
More specific and individualized dietary information about GDM	*“Pears are dangerous”*	Add diabetes-sensitive pictures;
	*“Other women with GDM can eat that, but not me”*	add features to register individual experiences with food items.
Design	*“I can’t see it”*	Enlarge pictures.
Presentation of blood sugar levels	*“I mean, I am a person who loves information provided in a graph”*	Provide feedback on individual blood sugar levels in a graph with graduated transitions and smileys.
	*“I would be scared if I would be in the red area”*	

Health information must be easily understandable and must meet the reader’s informational needs [32]. During our initial interviews, some participants had difficulty understanding some of the information about healthy eating and GDM. They also asked for explanations of medical terms. Given the increase in multi-cultural societies, there is a growing awareness that successful health information needs to address culture [33,34,35]. The prevalence of GDM in Norway is higher among immigrant women of African and Asian origins than in the majority population [36]. Therefore, we considered it important to make our app culturally sensitive. Resnicow *et al.* describe different dimensions of cultural sensitivity [37]. The first dimension of cultural sensitivity involves matching health information to observable characteristics of a target group. In our app, this involves translations to Urdu and Somali, using pictures of pregnant women various ethnic backgrounds and using food items familiar to and preferred in those cultures. In addition, we reviewed the app and applied Kreuter’s message checklist [38] and the Suitability Assessment of Materials [39] to improve the text and layout. We also developed a diabetes lexicon to explain medical jargon.

Our study followed the advice of several researchers, who stress that patients and health professionals should be involved directly in the design of smartphone health apps in order to increase effectiveness and usability [26,40,41,42]. Still, some patients were confused about information in the app that conflicted with advice from their health professionals. The patients’ confusion might indicate the need for an even tighter collaboration between app developers and healthcare professionals. Thus, we provided an additional round of quality improvement by reviewing written information from the clinics and discussing information inconsistencies with health professionals.

#### 3.2.2 Task Performance

In the second user-involvement study, participants were asked to perform six tasks on the second Pregnant+ prototype. Results must be interpreted with caution, as one participant forgot her glasses and had problems reading. Table 3 summarizes results of participants’ task performance. In general, participants had high scores on the main functions.

**Table 3:** Summary of successful task performance (%) with Pregnant+ prototype.

Task	Percentage of Participants Who Succeeded (*n* = 11)
Finding where to register blood sugar levels	100%
Finding information about healthy eating	91%
Finding inspiration for physical activity	91%
Finding information about gestational diabetes	91%
Entering appointments for medical consultations	82%
Finding how to register body weight	45%

### 3.3. Presentation of the Pregnant+ App

A review of mobile apps for diabetics highlights the importance of simple and understandable design, content, and menu navigation to development of a suitable medical device [26]. Involving a graphical user interface designer in development of our app and receiving feedback on design from patients were crucial.

#### 3.3.1. Individually Targeted Information

Results from discussions with researchers, health professionals and patients outlined the need for individually targeted app content. Individuals are more likely to actively process health information into behavior changes if they perceive the information as personally relevant [43,44]. Efforts in health communication vary in the degree to which they address individual characteristics [43]. Face-to-face communication between health professionals and patients provides the opportunity for the greatest degree of individualization. However, mobile technology can provide individually targeted health information [38]. To target the content of our app, users can choose to register the following information at first-time log-in and create their My Profile: (1) the outpatient clinic where they receive health care and the hospital where they will give birth; (2) their perceived level of physical activity before pregnancy; (3) their preferred food culture; and (4) their weight and height before pregnancy.

#### 3.3.2. Pregnant+ Main Content

**Figure 2:**
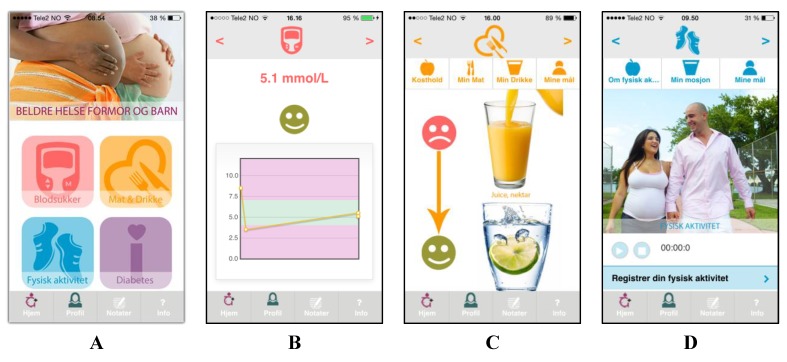
Presentation of the Pregnant+ app (shown on an iPhone interface): (**A**) four main icons (blood sugar, food and beverages, physical activity, diabetes) of the Pregnant+ smartphone app; (**B**) real-time feedback on blood sugar values; (**C**) information about healthy eating and drinking; and (**D**) information about physical activity.

The Pregnant+ app orders content hierarchically, using just four icons in order to avoid overloading the patient. Figure 2A presents these four icons. By selecting the first icon, “blood sugar”, patients can manually register or automatically transfer blood sugar levels to their smartphone when connected to the glucometer via Bluetooth. Providing feedback is an acknowledged method of providing individually targeted health information [45]. In our app, after measuring, patients receive immediate feedback on their blood sugar levels (Figure 2B) and targeted advice about healthy eating and physical activity. In addition, they can note what they ate prior to their blood sugar measurement. Patients can also generate a graph illustrating their blood sugar levels over time. Mobile apps with the capability to forward data to health professionals can provide significant advantages in diabetes health care [26]. Our users can print their registered blood sugar values at their clinics to discuss them with their health professional.

The second icon, “food and beverages”, provides information about a healthy diet for women with GDM. This section also provides information about food security and important nutrients during pregnancy. Pictures illustrating healthy eating are displayed according to a patient’s chosen food culture. How to eat healthily is demonstrated with pictures and the help of smileys (Figure 2C). Patients whose blood sugar measurements are too high will be immediately sent information about healthy eating and controlling blood sugar levels. In addition, the app links to recipes from the Norwegian Diabetes Foundation.

In the third icon, “physical activity”, patients can register the amount of time spent in physical activity and will receive feedback if they have met the recommendations of national health authorities (Figure 2D) [24]. In addition, they will receive information about physical activity that is tailored to their level of physical activity before pregnancy. Furthermore, the app provides illustrations of physical activity tailored to individual preferences.

The fourth icon, “diabetes”, provides general information about GDM and the diabetes lexicon. In addition, we developed a Question and Answer section based on patient interviews. In it, patients will find answers for initial worries and questions related to GDM.

#### 3.3.3. A Smartphone Application for Behavior Change

Developing GDM often means that women must change their diet and physical activity behaviors. Successfully guiding requires that the health information provided enables and motivates behavior changes. Most of the apps available for diabetes self-management have not been tested for effectiveness [18,19]. Behavior Change Techniques (BCTs) refer to specific strategies used in an intervention to promote behavior change. There is some evidence that the development of a smartphone app for women with GDM benefits from incorporating BCTs that are linked to relevant behavior change theories [46,47,48]. One effective BCT in these studies was the combination of self-monitoring with real-time feedback on users’ blood sugar levels. A review of smartphone apps to promote physical activity concluded that apps generally lack the use of BCTs [46]. Interventions can benefit from increasing risk awareness as a BCT [25]. Both health professionals and study participants asked for more information about the risks of GDM and the consequences for future health. Previous research has shown that women with GDM may have limited knowledge and risk awareness of the consequences of GDM [12,13,14,49]. It is generally acknowledged that insufficient risk and disease awareness are barriers to healthy behaviors and behavioral change that must be addressed when providing health information [25,50].

We identified the Health Belief Model (HBM) as an appropriate theory to guide the content of our app [25,51]. This theory has been considered relevant to pregnant women [52]. The theory suggests that in order to change behaviors, individuals need to be aware of their disease risk and perceive that the benefits of behavior change outweigh potential barriers [51]. The Pregnant+ app aims to contribute to behavior change by applying the following BCTs from the HBM: (I) perceived severity (information about consequences of risks and conditions related to GDM); (II) perceived benefits (information about the advantages of blood sugar control and a healthy lifestyle); and (III) cues to action (self-monitoring of blood sugar with real-time feedback and illustrations of how to eat healthily and be physically active).

## 4. Limitations

The Pregnant+ app will be translated into Somali and Urdu. Even though women from Somalia and Pakistan participated in the user-involvement studies, the app was presented only in the Norwegian language and the sample size was too small to extrapolate usability to different ethnic groups.

The number of participants in the user-involvement studies was limited. However, qualitative approaches gave useful insights in the first phase of designing and developing the Pregnant+ app. In addition, participants did not have the opportunity to test the automated transfer of blood sugar levels. This function was merely demonstrated in order to investigate their perceptions. A beta-test of the application will be performed in the feasibility study prior to the randomized controlled trial.

## 5. Conclusions

This paper contains details of developing a smartphone app for women with GDM. All relevant groups were involved in development, including health professionals, patients, data security and privacy experts and designers. The resulting app, Pregnant+, provides an automated transfer of measured blood sugar levels into the smartphone, which serves as a mobile health device for personalized diabetes care. Preserving privacy of medical data was a key requirement for the app, and privacy is ensured through keeping the blood sugar measurements in the device.

Close collaboration with health professionals while developing the smartphone app ensured that the mobile health device fits patients’ daily diabetes care. Patient perceived registration of blood sugar levels on the smartphone to be easier their current practice. In order to evaluate possible outcomes on blood sugar control and behavior change, this smartphone app will be tested in a randomized controlled trial involving five diabetes outpatient clinics. Health care professionals from these clinics were involved in the design and development process and will continue to provide consistent, up-to-date advice on nutrition, physical activity and GDM treatment.
